# The effects of gatekeeping on the quality of primary care in Guangdong Province, China: a cross-sectional study using primary care assessment tool-adult edition

**DOI:** 10.1186/s12875-019-0982-z

**Published:** 2019-07-04

**Authors:** Cuiying Liang, Jie Mei, Yuan Liang, Ruwei Hu, Li Li, Li Kuang

**Affiliations:** 1 Department of Health Administration, School of Public Health, Sun Yat-sen University, Guangzhou, 510080 China; 2 Department of Family Medicine and Community Health, Case Western Reserve University, Cleveland, Ohio 44106 USA; 3 Department of Family Medicine, University of Virginia, Charlottesville, Virginia USA

**Keywords:** Gatekeeping, Primary care, Quality of primary care, Propensity score matching, Cross-sectional survey

## Abstract

**Background:**

Developed countries have widely implemented a gatekeeping system as a core policy of primary care, also known as the system of first visit in the community. As gatekeepers, general practitioners are responsible for the diagnosis and treatment of residents in the community health centres, and referring patients to specialists as appropriate. After several years of healthcare reform, gatekeeping policy has achieved remarkable success in China. Shenzhen and Dongguan were the first batch of pilot cities that implemented the policy of gatekeeping. This study aims to examine the effects of gatekeeping on the quality of primary care between the gatekeeping and non-gatekeeping groups in these two pilot cities.

**Methods:**

A cross-sectional survey was conducted in five community health centres in Shenzhen and Dongguan cities, both located within Guangdong Province, China, using a validated Chinese version of the Primary Care Assessment Tool-Adult Edition (PCAT-AE) and carrying out face-to-face interviews with patients 18 years and older. Analyses were grouped according to whether or not patients had gatekeepers. Propensity Score Matching was used to control for confounding factors. A chi-square test was used to compare the factors mentioned above and an independent *t*-test was performed to compare the eight domains of the core functions of primary care between the two groups of patients.

**Results:**

In total, 765 valid questionnaires were collected for analysis, after matching the sample size were 238 pairs. All the confounding factors observed between the gatekeeping and non-gatekeeping groups were balanced. The PCAT-AE scores for first-contact utilisation (3.29 > 2.66, *p* < 0.001) and coordination (2.06 > 1.95, *p* < 0.05) were higher in the gatekeeping group after matching, but the domains of accessibility (1.59 < 1.67, *p* < 0.05) and continuity (2.26 < 2.40, *p* < 0.05) were lower. The PCAT-AE mean score was slightly higher in gatekeeping group (1.98 > 1.93, *p* > 0.05) but without statistical significance.

**Conclusion:**

This study demonstrated that gatekeeping has helped to improve first-contact utilisation and coordination of primary care, but that other goals such as continuity and comprehensiveness have been harmed. To establish a sustainable gatekeeping system and to strengthen the core functions of the community comprehensively, the current gatekeeping system needs refinement.

## Background

Many professional organisations propose a function-orientated definition of primary care consistent with the widely recognised multidimensional concept, including first contact, accessibility, continuity, comprehensiveness, and coordination, and three derivative dimensions including family centeredness, community orientation and cultural competence [1–3]. As a core policy of primary care, gatekeeping plays an important role in allocating health resources reasonably and maintaining the financial sustainability of the primary care system when facing population ageing and increases in prevalence of chronic conditions [4, 5]. General practitioners (GPs) are also gatekeepers. Many developed countries have adopted the policy of gatekeeping and it has been implemented widely in countries such as the United Kingdom and Switzerland [6–9].

The fundamental goal of gatekeeping is to strengthen basic primary care in the health service system; the principal functions of the primary care system will be realised by implementing basic public health services [10]. Gatekeeping requires patients to first visit their designated community health centre (CHC) and they need a GP’s referral before seeking care at specialty care or hospital facilities [11]. As such, gatekeeping has a clinical function where patients use GPs as an entry point into medical care [12], it thus protects patients from the possible adverse effects of unnecessary care [13]. This policy has proven that it can increase first contact and provide better coordination of care [14–16] to ensure equity by matching healthcare needs and healthcare services including specialty referrals [15, 17]. The gatekeepers identify patients’ healthcare needs and choose services effectively to meet or address patients’ needs, providing an important filter to specialist care [18].

In 2009, China officially announced the *Instruction of the CPC Central Committee and State Council on Deepening the Reform of the Medical and Health Care System* [19] that strengthened delivery of community-based primary care, established a referral system [20] and expanded the role of CHCs as the first contact of care and as gatekeepers to the entire health system [21]. China has made remarkable achievements since 2009. First, the primary care system successfully achieved universal medical insurance coverage for 97.5% of the Chinese population in 2014 [22–24]. The government also increased subsidies for CHCs [10], which increased the affordability of primary care and economic accessibility [24]. Second, the government expanded and improved the network of primary care facilities, 84.0% residents could reach CHCs within 15 min in 2013 [25], which improved geographic accessibility [26]. Third, the service capacity of CHCs was strengthened. The government instituted a national essential drug system [27] and a basic public health service programme. CHCs provided low-cost drugs and free basic public health services for residents [28], including establishing health records, chronic disease management and so on, which improved access to and availability of primary health services [10].

Guangdong Province is considered as a healthcare policy trendsetter, which has played a leading role in well-establishing models of primary care [29]. Shenzhen and Dongguan were the first batch of pilot cities to implement the policy of gatekeeping, in 2006 and 2008 respectively [7, 20]. These were both developed cities, with large number of laborers, located in Guangdong Province, the Pearl River Delta region in southern China. To ensure equity of the health service, the basic medical insurance institution was formed which covered the migrant population, and urban and rural residents. To explore ways to strengthen primary care service systems when financial resources were limited, the gatekeeping policy was adopted. The governments of Shenzhen and Dongguan have established social health insurance with a low financing level and achieved universal health coverage through the implementation of gatekeeping system, especially guaranteeing the health of the vulnerable migrant population.

The governments used insurance payment mechanisms to require patients to use CHCs as their first contact within the compulsory gatekeeping system [30]. In addition to emergency service, a person participating in social health insurance should choose CHCs first. The Shenzhen social insurance department calculated the annual compensation from the number of people registered in the CHCs and the standard of the overall pooling of outpatients. In this system, although those insured were at a low payment level (12 yuan per person per month), the reimbursement rate was nearly 80%. The Shenzhen government subsidised basic public health services for the CHCs at 40 yuan per service population in 2010. Dongguan has successfully implemented a comprehensive and integrated medical insurance system covering workers and residents, and formed a unified social pooling fund for health care, which has had a unified payment and compensation standards since 2008. An insured person would pay 10 yuan per person per month for the insurance system, with a reimbursement rate of up to 70% for outpatient treatment in CHCs. These two cities both face the challenge of how to meet the basic health demands of the residents with limited resources and economic constraints. In this situation, reducing and controlling medical expenses became the inevitable choice of the governments.

However, most previous evaluations of the effect of gatekeeping have focused on health outcomes, quality of life, economic outcomes, healthcare utilisation (e.g. hospitalisation, and specialist and emergency services) and patients’ satisfaction [11, 31–33]. In the primary care system, cost containments mainly come from the function of coordination, which acts as a filter, and from making better use of specialist resources [34]. The functions of first contact and continuity will reduce the utilisation of hospitalisation and specialist services [34, 35]. The functions of accessibility and comprehensiveness can improve patients’ general overall healing and health outcomes such as quality of life [15, 34]. Lastly, patients’ satisfaction will be improved. The benefit are a result of the combined effect of the functions of first contact, accessibility, coordination, continuity and comprehensiveness [16, 35]. The core functions of primary care are the driving force that leads to primary care playing an important role in containing health expenditures, reducing the utilisation of hospitalisation and specialist services, and improving the patients’ quality of life and satisfaction. Therefore, evaluating gatekeeping from the perspective of the core functions of primary care will provide more comprehensive and targeted evidence for policymaking. We adopted the Donabedian model which describes the primary care process determined by the core and derivative dimensions. The quality of primary care as the outcome can be measured through primary care’s functions [36].

The available evidence indicates that gatekeeping is related to lower utilisation of health services and lower expenditures but not to an increase in patients’ satisfaction [11, 31, 32, 37, 38]. However, to our knowledge, there has been no study assessing gatekeeping from the angle of the core functions of primary care in China. Our study examined the effects of gatekeeping on the quality of primary care in the first batch of pilot cities in Guangdong Province, China by using the Primary Care Assessment Tool -Adult edition (PCAT-AE). We adopted Propensity Score Matching (PSM) to effectively adjust for confounders and to facilitate comparability between the two groups in our research. The significance of this research is that it aimed to discover any vulnerable points of the policy to provide evidence for advancing a wider range of gatekeeping systems and for perfecting the healthcare system.

## Methods

### Population and sample

A cross-sectional survey was conducted in Shenzhen and Dongguan to investigate their implementation of the gatekeeping policy. We used a stratified, two-stage sampling approach in our study. In the first stage, we used purposive sampling and selected three CHCs in Shenzhen and two in Dongguan. These CHCs were selected due to the large number of outpatient visits. In the second stage, we used convenience sampling to select participants in each CHC. We recruited patients whose usual source of primary care was the study site. The inclusion criteria for the participants were as follows: (1) patients who were 18 years or older; (2) patients who could speak Mandarin or Cantonese clearly in the waiting area of each site; and (3) patients who had visited the same CHCs or GPs at least three times, in order to guarantee that they had a better understanding of primary care services. The exclusion criteria were as follows: (1) those who were in poor physical condition and could not complete the questionnaire; and (2) those who had trouble understanding the questionnaire. Based on the standard sample size formula for a cross-sectional study, a target sample size of 400 was set for each city, given a type I error of 0.05, type II error of 0.1, and refusal rate of 10% [39].

### Data collection

The study data collection began in June 2014 and ended in August 2014. The interviewers were four postgraduate students from Sun Yat-sen University. They were trained in advance by two researchers so that they could assist the patients to complete the questionnaires. To guarantee the survey’s quality, one-to-one and face-to-face interviews were implemented in the waiting area. The potential participants were asked for permission to participate in the interview before their GP’s visits and after a full explanation of the research purpose. We declared that the survey would not influence their usual visits to GPs. After completing the questionnaire, they received a small gift as a token of our appreciation. All participants provided verbal consent. The Institutional Review Board of Sun Yat-sen University reviewed and approved this method of obtaining verbal consent from patients.

### Measures

We used an internationally recognised assessment tool called the PCAT-AE to assess quality of primary care. This instrument had been originally developed by John Hopkins for use in the USA [40]. It has since been adapted in many other countries including China [23, 41, 42]. The PCAT-AE is an instrument with good reliability and validity in China [23, 39, 40]. The accumulative variance is 58.91% and the overall Cronbach’s α is 0.74 [23]. The PCAT-AE measures the five core dimensions of primary care including first contact, accessibility, continuity, comprehensiveness, and coordination and three derivative dimensions—family centeredness, community orientation and cultural competence [34, 43]. Forty-two items were included in the Chinese version of the questionnaire: 25 items assessed eight dimensions of primary care, 1 item identified types of insurance, 2 items identified individuals’ usual source of care, 2 items measured the frequency of visits to GPs, 1 item assessed patients’ total satisfaction about their current provider of health care, and 8 items reflected patients’ socioeconomic characteristics. The remaining items were used to reflect the patient’s physical condition and identified whether or not the patient was contracted with a GP. The services received by patients were represented using a 4-point Likert scale (1 = never; 2 = sometimes; 3 = often; 4 = always). An additional option of ‘Don’t know/Not sure’ was added to prevent lack of knowledge on a certain item. This option was assigned 2.5 as a neutral value when conducting our analyses to be consistent with methods used in China [39] and other countries [41, 42].

### Data analysis

Ultimately, 767 participants completed the questionnaire with a refusal rate of 4%. Two were dropped from the analysis due to missing key values. We divided the participants into two groups (gatekeeping and non-gatekeeping) according to their type of insurance. The regulations of medical insurance in Shenzhen require residents with type II and type III insurance to be subject to the gatekeeping group. Those with other insurance or no insurance were subject to non-gatekeeping group. According to the reimbursement standard in Dongguan, those with Urban Resident Basic Medical Insurance were the gatekeeping group. Those with Urban Employee Basic Medical Insurance or no insurance were the non-gatekeeping group. Continuous variables were presented as the mean ± standard deviation, and categorical variables were presented as frequency (%). PSM was employed through a nearest neighbor-matching algorithm, with a match tolerance of 0.01. Some previous studies have shown that patients’ socioeconomic characteristics, utilisation of healthcare and health status [11, 30, 44] and the location of CHCs have significant impacts on their PCAT scores. The type of insurance, however, has been shown to have no effect on it [45]. PSM was used to control for influencing factors. The propensity score was constructed using a common logistic regression model, in which potential confounding variables were considered as independent variables, including city location, gender, age, household registration status, household income, marital status, education, working status, whether or not contracted with a GP, self-perceived health status, chronic condition, period of time since their first visit and number of GP visits in the past year. Group assignment was included as the dependent variable. After matching, we examined whether the factors were matched smoothly. We used a chi-square test to compare socioeconomic characteristics, utilisation of healthcare and health status between the two groups. In addition, independent *t*-tests were applied to compare primary care attribute scores reported by the two groups before and after matching. The level of significance was *p* < 0.05. All data analysis was performed using IBM SPSS Statistics version 22.0.

## Results

### Socioeconomic characteristics of the participants

In total, 765 questionnaires were eligible for analysis before PSM, including 356 participants in the ‘non-gatekeeping’ group and 409 participants in the ‘gatekeeping’ group. There were 238 pairs after PSM. Table 1 presents the frequency and percentage distribution according to socioeconomic characteristics, utilisation of community health care, and health status between the two groups of participants before and after PSM. Overall before PSM, there were many significant differences in characteristics between the gatekeeping and non-gatekeeping groups. In both groups, most of the respondents were migrants, married, employed, not contracted with a GP, with an excellent or good health status and without chronic conditions. More participants had gatekeepers than did not (53.5% > 46.5%). The non-gatekeeping group appeared to have a higher percentage of women (68.0% > 49.4%, *p* < 0.001), were younger (37.6% > 28.6%, *p* < 0.05), richer (31.5% > 24.0%, *p* < 0.05), more educated (29.2% > 18.8%, *p* < 0.01) and had a higher rate of unemployment (27.0% > 13.4%, *p* < 0.001). In terms of health status, the gatekeeping group were more likely to have a better self-perception of their health conditions (76.0% > 66.9%, *p* < 0.01). The number of GP visits in the past year in the gatekeeping group was more than in the non-gatekeeping group (7.6% > 4.5%, *p* < 0.05). All the confounding factors displayed in Table 1 had a balanced match after PSM.

**Table 1:** Sample characteristics of participants with and without gatekeeping before and after PSM

	Before Matching				After Matching			
	Non-gatekeeping N (%)	Gatekeeping N (%)	Total, N (%)	*P*	Non-gatekeeping N (%)	Gatekeeping N (%)	Total N (%)	*P*
Sample size	356 (46.5)	409 (53.5)	765 (100)		238 (50)	238 (50)	476 (100)	
Cities								
Dongguan	135 (37.9)	264 (64.5)	399 (52.2)	**< 0.001**	124 (52.1)	118 (49.6)	242 (50.8)	0.647
Shenzhen	221 (62.1)	145 (35.5)	366 (47.8)		114 (47.9)	120 (50.4)	234 (49.2)	
Gender								
Male	114 (32.0)	207 (50.6)	321 (42.0)	**< 0.001**	99 (41.6)	92 (38.7)	191 (40.1)	0.575
Female	242 (68.0)	202 (49.4)	444 (58.0)		139 (58.4)	146 (61.3)	285 (59.9)	
Age								
≤30	134 (37.6)	117 (28.6)	251 (32.8)	**0.026**	82 (34.5)	84 (35.3)	166 (34.9)	0.354
31–60	205 (57.6)	273 (66.7)	478 (62.5)		144 (60.5)	148 (62.2)	292 (61.3)	
> 60	17 (4.8)	19 (4.6)	36 (4.7)		12 (5.0)	6 (2.5)	18 (3.8)	
Migrant								
No	80 (22.5)	87 (21.3)	167 (21.8)	0.726	45 (18.9)	54 (22.7)	99 (20.8)	0.366
Yes	276 (77.5)	322 (78.7)	598 (78.2)		193 (81.1)	184 (77.3)	377 (79.2)	
Household monthly income								
< 5000	89 (25.0)	128 (31.3)	217 (28.4)	**0.036**	65 (27.3)	60 (25.2)	125 (26.3)	0.870
5000–10,000	155 (43.5)	183 (44.7)	338 (44.2)		111 (46.6)	115 (48.3)	226 (47.5)	
> 10,000	112 (31.5)	98 (24.0)	210 (27.5)		62 (26.1)	63 (26.5)	125 (26.3)	
Marital status								
Married	318 (89.3)	350 (85.6)	668 (87.3)	0.128	207 (87.0)	203 (85.3)	410 (86.1)	0.691
Not married	38 (10.7)	59 (14.4)	97 (12.7)		31 (13.0)	35 (14.7)	66 (13.9)	
Education								
Primary school or lower	38 (10.7)	40 (9.8)	78 (10.2)	**0.002**	30 (12.6)	19 (8.0)	49 (10.3)	0.173
Middle/High school	214 (60.1)	292 (71.4)	506 (66.1)		147 (61.8)	163 (68.5)	310 (65.1)	
College or above	104 (29.2)	77 (18.8)	181 (23.7		61 (25.6)	56 (23.5)	117 (24.6)	
Working status								
Employed	245 (68.8)	345 (84.4)	590 (77.1)	**< 0.001**	185 (77.7)	192 (80.7)	377 (79.2)	0.142
Retired	15 (4.2)	9 (2.2)	24 (3.1)		10 (4.2)	3 (1.3)	13 (2.7)	
Unemployed	96 (27)	55 (13.4)	151 (19.7)		43 (18.1)	43 (18.1)	86 (18.1)	
Contracted with a GP								
No	337 (94.7)	393 (96.1)	730 (95.4)	0.388	229 (96.2)	226 (95.0)	455 (95.6)	0.656
Yes	19 (5.3)	16 (3.9)	35 (4.6)		9 (3.8)	12 (5.0)	21 (4.4)	
Self-perceived health status health status								
Excellent/Very good/good	238 (66.9)	311 (76.0)	549 (71.8)	**0.006**	166 (69.7)	164 (68.9)	330 (69.3)	0.921
Poor/Fair	118 (33.1)	98 (24.0)	216 (28.2)		72 (30.3)	74 (31.1)	146 (30.7)	
Chronic condition								
No	276 (77.5)	288 (70.4)	564 (73.7)	**0.026**	184 (77.3)	181 (76.1)	365 (76.7)	0.828
Yes	80 (22.5)	121 (29.6)	201 (26.3)		54 (22.7)	57 (23.9)	111 (23.3)	
Period of time since the first visit								
< 2 Years	153 (43.0)	197 (48.2)	350 (45.8)	0.309	103 (43.3)	117 (49.2)	220 (46.2)	0.225
2–5 Years	100 (28.1)	110 (26.9)	210 (27.5)		70 (29.4)	54 (22.7)	124 (26.1)	
> 5 Years	103 (28.9)	102 (24.9)	205 (26.8)		65 (27.3)	67 (28.2)	132 (27.7)	
Number of GP visits in the past year								
< 3	136 (38.2)	122 (29.8)	258 (33.7)	**0.041**	118 (49.6)	91 (38.2)	209 (43.9)	0.064
3–5	125 (35.1)	149 (36.4)	274 (35.8)		72 (30.3)	79 (33.2)	151 (31.7)	
6–15	79 (22.2)	107 (26.2)	186 (24.3)		40 (16.8)	57 (23.9)	97 (20.4)	
> 15	16 (4.5)	31 (7.6)	47 (6.1)		8 (3.4)	11 (4.6)	19 (4.0)	

*N* number of participants, *GP* general practitioner

*P* values were based on chi-square test of difference between those with gatekeepers and those without. Significance indicated at *p *< 0.05

### Propensity score matching (PSM) results

**Fig. 1:**
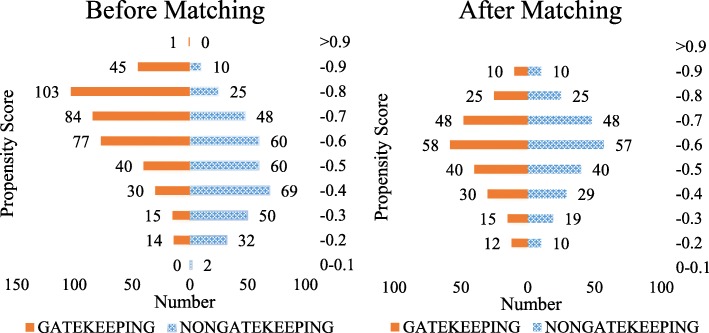
Distribution of propensity scores before and after matching between two groups

Figure 1 shows the distribution of propensity scores before and after matching between the two groups. We found that the differences of propensity scores between ‘gatekeeping’ and ‘non-gatekeeping’ were almost balanced after matching.

### Quality of primary care scores

**Fig. 2:**
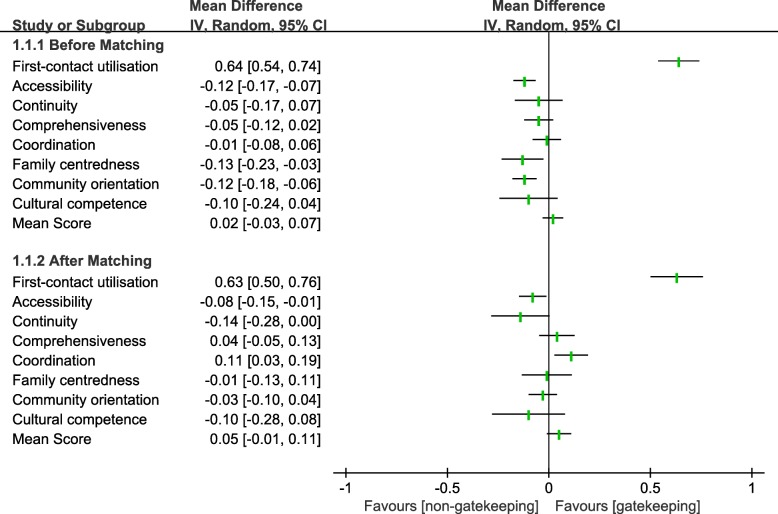
Analysis of primary care attributes scores between two groups before and after PSM

Figure 2 shows the forest map of the scores of primary care attributes using eight quality indicators before and after PSM. The mean difference is shown as the mean of the score of the gatekeeping minus non-gatekeeping groups. The mean PCAT-AE score is the mean of the eight domain scores and reflects an overall measure of quality of primary care. Table 2 shows the comparison between the two groups in more detail. In general, compared with the non-gatekeeping group, the mean PCAT-AE scores of the gatekeeping group was a little higher than the non-gatekeeping group before (1.99 > 1.97, *p* > 0.05) and after PSM (1.98 > 1.93, *p* > 0.05), but without statistical significance. However, data from Table 2 indicates that, before PSM gatekeeping only performed better than non-gatekeeping in first-contact utilisation (3.31 > 2.67, *p* < 0.001). The score of primary care was statistically significant between the gatekeeping and non-gatekeeping participants in the domains of first-contact utilisation, accessibility, family centeredness and community orientation (*p* < 0.05). After PSM, Table 2 shows that the score of first-contact utilisation (3.29 > 2.66, *p* < 0.001) and coordination (2.06 > 1.95, *p* < 0.05) in the gatekeeping group was substantially higher than in the non-gatekeeping group. Surprisingly, the domains of accessibility and continuity showed the opposite. The scores of accessibility (1.67 > 1.59, *p* < 0.05) and continuity (2.40 > 2.26, *p* < 0.05) were higher in the non-gatekeeping group. There were some differences in other domains but they did not achieve statistical significance (*p* > 0.05). In terms of the differences between the two types of settings, the participants in both groups had no difference in the domain of degree of satisfaction before or after PSM (*p* > 0.05).

**Table 2:** Scores of primary care attributes and satisfaction between two groups before and after PSM

	Gatekeeping mean (SD)	Non-gatekeeping mean (SD)	*P*
Before Matching			
Mean score	1.99 (0.33)	1.97 (0.38)	0.675
First contact-utilisation*	3.31 (0.68)	2.67 (0.74)	< 0.001
Accessibility*	1.59 (0.39)	1.71 (0.38)	< 0.001
Continuity	2.35 (0.86)	2.40 (0.80)	0.416
Comprehensiveness	1.69 (0.47)	1.74 (0.53)	0.174
Coordination	2.01 (0.46)	2.02 (0.52)	0.836
Family centredness*	1.72 (0.66)	1.85 (0.78)	0.018
Community orientation*	1.20 (0.33)	1.32 (0.48)	< 0.001
Cultural competence	1.99 (1.02)	2.09 (1.00)	0.195
Degree of satisfaction	3.70 (0.68)	3.75 (0.71)	0.939
After matching			
Mean score	1.98 (0.32)	1.93 (0.35)	0.097
First contact-utilisation*	3.29 (0.70)	2.66 (0.74)	< 0.001
Accessibility *	1.59 (0.39)	1.67 (0.36)	0.013
Continuity*	2.26 (0.80)	2.40 (0.80)	0.047
Comprehensiveness	1.72 (0.47)	1.68 (0.50)	0.447
Coordination*	2.06 (0.43)	1.95 (0.49)	0.011
Family centredness	1.74 (0.65)	1.75 (0.72)	0.987
Community orientation	1.23 (0.33)	1.26 (0.44)	0.494
Cultural competence	1.98 (1.00)	2.08 (1.00)	0.273
Degree of satisfaction	3.69 (0.69)	3.70 (0.70)	0.843

*SD* Standard Deviation

*Significance indicated at *p* < 0.05, based on *t*-test of difference between those with gatekeepers and those without

**Fig. 3:**
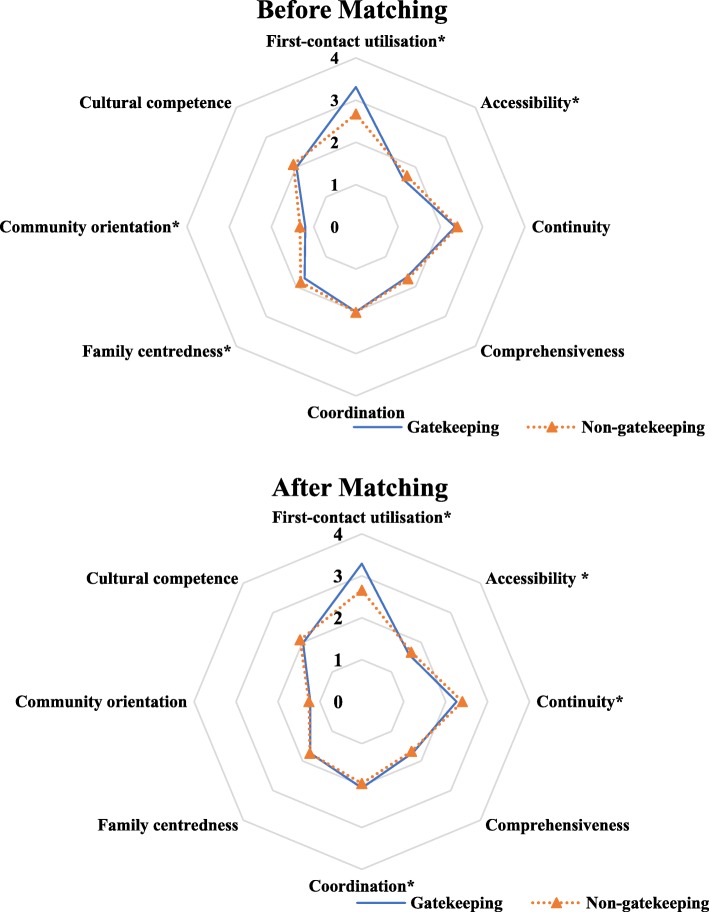
Scores of primary care attributes between patients with and without gatekeeping before and after PSM

The radar chart shown in Fig. 3 provides more detail about the scores of primary care attributes reported by the gatekeeping and non-gatekeeping participants before and after PSM. The score gap between the two groups is clear in each domain. Figure 3 exhibits a large gap between the two groups in the domain of first-contact utilisation before matching. Before matching, we found that the non-gatekeeping group obviously higher scores in the domain of accessibility, family centeredness and community orientation. There were no differences in the domains of continuity, comprehensiveness, coordination and cultural competence. After matching, the largest difference was also in the domain of first-contact utilisation. Those in the gatekeeping group reported higher scores in the domains of continuity and coordination. The scores of family centeredness and community orientation between the two groups became closer so that there appeared to be no difference. The other domains showed a similar pattern to that before matching.

## Discussion

In this cross-sectional study, a validated Chinese version of PCAT-AE was used to assess the effects of gatekeeping on the quality of primary care in CHCs in Shenzhen and Dongguan cities, China, in 2014. Our findings indicate that the policy of compulsory gatekeeping was associated with greater improvement in quality of primary care in some domains while declining in others.

We found that the score of first-contact utilisation was significantly higher in the gatekeeping group, which was consistent with other studies [7, 11]. Forrest suggested that gatekeeping increased levels of first-contact care by altering patients’ behaviour [18]. This is related to the implementation of the first contact in CHCs compulsory policy in these two cities. The insurance system requires that patients make CHCs their first contact of care when new health problems emerge. This could reduce the reimbursement rate for using health services in the tertiary hospitals without referrals from designated CHCs. To their credit, the governments of Shenzhen and Dongguan resolved most of the residents’ health issues with only a small amount of money. It is obvious that Shenzhen and Dongguan’s compulsory gatekeeping policy played a role in guiding patients to seek community healthcare services first.

Our results also show that the participants in the gatekeeping group experienced higher performance in the coordination domain, which was consistent with other findings [31, 46]. These patients used primary care as first contact, thus GPs had more opportunities to provide more coordinating services for them, which could have improved coordination [15]. In addition, the performance in this domain was related to insurance regulations. These stipulate that those needing to be referred to hospital usually require a physician referral and reason for referral in their medical records. In the coordination dimension of our study, the score for the items ‘Did your GP discuss with you different places you could have gone to get help with that problem? / Did your doctors write down any information for the specialist about the reason for that visit?’ were both higher in the gatekeeping group.

Paradoxically, there was no positive impact on the other domains of primary care in the gatekeeping group. In our study, the participants in gatekeeping group had lower scores in the domain of accessibility and continuity. Since the implementation of the compulsory gatekeeping policy, a large number of patients have crowded into the CHCs, highlighting the shortage of GPs. According to *The China Health and Family Planning Statistical Yearbook 2015* [47], each GP was responsible for 7200 inhabitants in Guangdong Province, which can be compared with 2000 in Portugal [48], 1500 in England [49] and 2100 in the USA [50]. Substantial shortages of GPs exist in China, which led to the longer waiting times to visit GPs found in this study. This is a logical organisational response to the scarcity of physicians within such a system. Conversely, patients in the non-gatekeeping group were free to choose facilities, so they visited the CHCs when they though CHCs would be satisfactory and convenient for them. In our study, the score for the item ‘Once you get there, do you have to wait for more than 15 minutes before you are checked by the doctor or nurse?’ was lower in the gatekeeping group. Studies have shown that countries with a gatekeeping policy had an average longer wait of days for an appointment than those without, due to the limited supply of physicians [18, 51]. Li’s study suggested that patients with gatekeepers were less satisfied with waiting times than those without gatekeepers [11]. This suggests that increasing the number of GPs would ensure an adequate supply and improve the access to primary care. Another explanation is that patients under gatekeeping were more likely to expect shorter waiting time, so they tended to exaggerate the waiting time according to the investigation on site. This may explain why the gatekeeping group scored lower than the non-gatekeeping group in the domain of accessibility.

In the dimension of continuity, there are several reasons for the lower score in the gatekeeping group: first, the policy of gatekeeping requires that patients visit the CHCs where they registered. However, the patients do not have a specific GP responsible for establishing an one-on-one relationship with them, leading to an inability to establish a continuous relationship between GP and patient. Second, Forrest’s study revealed that a longer waiting time and lack of after-hours care were associated with lower levels of continuity [52]. This means that, the access to primary care will influence the likelihood of patients establishing a relationship with GPs, thus affecting continuity of care [15, 34]. Third, in the setting of this investigation, most CHCs use a ‘patients calling system’ in the outpatient waiting area and patients are randomly assigned to a doctor on a first-come-first-served basis, so that patients cannot request the same GP to provide their health service. Halm [53], Grumbach [54], and Shi [55] pointed out that the negative effect of gatekeeping was probably related to perceived adverse influences on the physician-patient relationship. They suggested that gatekeepers undermined patients’ trust and confidence because of impeding access to specialists. What have been discussed above may explain why the gatekeeping group scored lower than the non-gatekeeping group in this domain. In response to dealing with an increasing number of people due to the gatekeeping policy, CHCs need to modify the structure to facilitate accessibility and coordination of care [12]. Moreover, continuity of care is associated with better comprehensiveness [35]. This suggests that communities should speed up the construction of supporting facilities and optimise the service process to promote the implementation of the core functions of primary care. While establishing the system of first contact in registered CHCs, GPs in the CHCs should be required to be responsible for establishing an one-on-one relationship with patients to strengthen continuity.

There was no difference between the two groups in the dimension of comprehensiveness or the three derivative dimensions. This is probably because the community physicians still provided the traditional one-visit-based model of healthcare service, focused on the disease model of medical care; therefore, the basic health services were not integrated at the individual level. The practice model of GPs in China has not yet been able to evolve into a patient-centred service model [56], which focuses on the holistic concept of time, space and therapies. At present, the government attaches great importance to the basic public health service and is committed to enriching the community service, however, evaluating from the patient’s point of view, the score of comprehensiveness is still a little low. There was also no increase in patient satisfaction about their care at CHCs with the gatekeeping policy, which was consistent with previous studies [33, 38].

More importantly, the investment of resources is required to realise the core functions of primary care, including accessibility, continuity and comprehensiveness. The development of CHCs greatly depends on support for social resources. However, in our study, the insurance payment level was low. This is probably because the actual level of expenses compensated by the governments of Shenzhen and Dongguan was too low, so that CHCs were not able to take on the responsibility for providing so many basic services in such a service pattern to meet the residents’ demands. It makes sense that gatekeeping alone cannot increase patients’ satisfaction or the quality of primary care, but the insurance system should pay attention to the sustainability of the gatekeeping system in the future. The focus should be not just on cost containment but on strengthening the construction of the core functions of primary care, increasing the payment level to the community and ensuring that the community has the resources to achieve the core functions noted above. The government should increase the compensation of health insurance and the capitation fees of the patients to ensure adequate resources support for primary care. A good gatekeeping policy should ultimately balance clinical needs, patient choice and system constraints [57].

There are several limitations to consider for this study. First, the study sites were selected from only two cities due to the pilot nature of the gatekeeping policy, which limited its promotion to other cities. More experiences are needed to generalise the results to other regions. Second, although we controlled possible confounding factors when comparing the quality of primary care between the two groups by using PSM, other unmeasured confounders may not have been identified and controlled, such as psychological factors which could influence a patient’s choice. Despite these limitations, this study used PSM innovatively to balance the confounding factors so as to examine the effect of gatekeeping on the quality of primary care. In addition, the findings from this study are useful in informing both policy decisions and practice. They provide valuable evidence that the role of gatekeeping can enhance the quality of primary care. This research would not only inform policymakers to promote the implementation and dissemination of the gatekeeping policy in China, but also in other developing countries.

## Conclusions

Our study demonstrated that gatekeeping had improved first-contact utilisation and coordination, which provides a basis for policymakers to promote the implementation of the gatekeeping system. Other goals of the policy, however, such as accessibility and continuity have not yet been achieved. To establish a sustainable gatekeeping system and to strengthen the core functions of the community comprehensively, the current gatekeeping system of primary care service needs refinement. The government should vigorously promote first contact, perfect the system of gatekeeping, establish supporting policies and measures, which must also be adaptable and anticipatory of future requirements and strengthen the construction of primary care and the community public service functions. The government should also give more support through resources.

## Data Availability

Please contact the corresponding author for data requests.

## Abbreviations

CHC: Community health centre
GP: General practitioner
PCAT-AE: Primary Care Assessment Tool-Adult Edition
PSM: Propensity Score Matching

## References

[1] Institute of Medicine Committee on the Future of Primary Care (1994). Defining primary care: an interim report.

[2] Wonca European (2005). The European Definition of General Practice/Family Medicine.

[3] World Health Organization (1988). From Alma Ata to the year 2000: reflections at the midpoint.

[4] Franks P, Clancy CM, Nutting PA (1992). Gatekeeping revisited--protecting patients from overtreatment. N Engl J Med.

[5] Xu J, Mills A (2017). Challenges for gatekeeping: a qualitative systems analysis of a pilot in rural China. Int J Equity Health.

[6] Schwenkglenks M, Preiswerk G, Lehner R, Weber F, Szucs TD (2006). Economic efficiency of gatekeeping compared with fee for service plans: a Swiss example. J Epidemiol Community Health.

[7] Gan Yong, Li Wenzhen, Cao Shiyi, Dong Xiaoxin, Li Liqing, Mkandawire Naomie, Chen Yawen, Herath Chulani, Song Xingyue, Yin Xiaoxv, Yang Tingting, Li Jing, Deng Jian, Lu Zuxun (2016). Patients’ Willingness on Community Health Centers as Gatekeepers and Associated Factors in Shenzhen, China. Medicine.

[8] Gervas J, Perez Fernandez M, Starfield BH (1994). Primary care, financing and gatekeeping in western Europe. Fam Pract.

[9] van Loenen T, van den Berg MJ, Heinemann S, Baker R, Faber MJ, Westert GP (2016). Trends towards stronger primary care in three western European countries; 2006-2012. BMC Fam Pract.

[10] Li X, Lu J, Hu S, Cheng KK, De Maeseneer J, Meng Q (2017). The primary health-care system in China. Lancet (London, England).

[11] Li Wenzhen, Gan Yong, Dong Xiaoxin, Zhou Yanfeng, Cao Shiyi, Kkandawire Naomiem, Cong Yingjie, Sun Huilian, Lu Zuxun (2017). Gatekeeping and the utilization of community health services in Shenzhen, China. Medicine.

[12] Forrest CB, Nutting P, Werner JJ, Starfield B, von Schrader S, Rohde C (2003). Managed health plan effects on the specialty referral process: results from the ambulatory sentinel practice network referral study. Med Care.

[13] Aoki Takuya, Yamamoto Yosuke, Ikenoue Tatsuyoshi, Kaneko Makoto, Kise Morito, Fujinuma Yasuki, Fukuhara Shunichi (2018). Effect of Patient Experience on Bypassing a Primary Care Gatekeeper: a Multicenter Prospective Cohort Study in Japan. Journal of General Internal Medicine.

[14] Flynn KE, Smith MA, Davis MK (2002). From physician to consumer: the effectiveness of strategies to manage health care utilization. Med Care Res Rev.

[15] Sommers AR, Wholey DR (2003). The effect of HMO competition on gatekeeping, usual source of care, and evaluations of physician thoroughness. Am J Manag Care.

[16] Starfield B, Shi L, Macinko J (2005). Contribution of primary care to health systems and health. Milbank Q.

[17] Ang KT, Ho BK, Mimi O, Salmah N, Salmiah MS, Noridah MS (2014). Factors influencing the role of primary care providers as gatekeepers in the Malaysian public healthcare system. Malays fam physician.

[18] Forrest CB (2003). Primary care in the United States - primary care gatekeeping and referrals: effective filter or failed experiment?. Br Med J.

[19] Chen Z (2009). Launch of the health-care reform plan in China. Lancet.

[20] Shi L, Lee DC, Liang H, Zhang L, Makinen M, Blanchet N (2015). Community health centers and primary care access and quality for chronically-ill patients - a case-comparison study of urban Guangdong Province, China. Int J Equity Health.

[21] Xu J, Wang W, Li Y, Zhang J, Pavlova M, Liu H (2010). Analysis of factors influencing the outpatient workload at Chinese health centres. BMC Health Serv Res.

[22] Yu H (2015). Universal health insurance coverage for 1.3 billion people: what accounts for China's success?. Health policy.

[23] Mei J, Liang Y, Shi L, Zhao J, Wang Y, Kuang L (2016). The development and validation of a rapid assessment tool of primary Care in China. Biomed Res Int.

[24] Xiong X, Zhang Z, Ren J, Zhang J, Pan X, Zhang L (2018). Impact of universal medical insurance system on the accessibility of medical service supply and affordability of patients in China. PLoS One.

[25] National Health Commission of the People’s Republic of China. An Analysis Report of National Health Services Survey in China, 2013. The government of the People's Republic of China. 2016. http://www.nhc.gov.cn/mohwsbwstjxxzx/s8211/201610/9f109ff40e9346fca76dd82cecf419ce.shtml. Accessed 25 June 2019.

[26] Zhao C, Wang C, Shen C, Wang Q (2018). China's achievements and challenges in improving health insurance coverage. Drug Discov Ther.

[27] The government of the People's Republic of China. National Essential Medicines List (2012 Edition) (Decree no. 93 by the Ministry of Health). 2013. http://www.gov.cn/gzdt/2013-03/15/content_2355142.htm. Accessed 25 June 2019.

[28] National Health Commission of the People’s Republic of China. The Notice on Strengthening the Work of the 2017 National Basic Public Health Service Project. The government of the People's Republic of China. 2017. http://www.nhc.gov.cn/xxgk/pages/viewdocument.jsp?dispatchDate=&staticUrl=/jws/s3577/201709/fb16b2e306bd469ab84e0c42173bc52d.shtml. Accessed 25 June 2019.

[29] Hu R, Liao Y, Du Z, Hao Y, Liang H, Shi L. Types of health care facilities and the quality of primary care: a study of characteristics and experiences of Chinese patients in Guangdong Province, China. BMC Health Serv Res. 2016;16(a):335. 10.1186/s12913-016-1604-2 PMC4969734 27484465

[30] Kuang L, Liang Y, Mei J, Zhao J, Wang Y, Liang H (2015). Family practice and the quality of primary care: a study of Chinese patients in Guangdong Province. Fam Pract.

[31] Velasco Garrido M, Zentner A, Busse R (2011). The effects of gatekeeping: a systematic review of the literature. Scand J Prim Health Care.

[32] Zentner A, Velasco MG, Busse R (2010). Do primary care physicians acting as gatekeepers really improve health outcomes and decrease costs? A systematic review of the concept gatekeeping. Gesundheitswesen.

[33] Wu J, Zhang S, Chen H, Lin Y, Dong X, Yin X (2016). Patient satisfaction with community health service centers as gatekeepers and the influencing factors: a cross-sectional study in Shenzhen, China. PLoS One.

[34] Kringos DS, Boerma WG, Hutchinson A, van der Zee J, Groenewegen PP (2010). The breadth of primary care: a systematic literature review of its core dimensions. BMC Health Serv Res.

[35] Starfield B (2012). Primary care: an increasingly important contributor to effectiveness, equity, and efficiency of health services. SESPAS report 2012. Gac Sanit.

[36] Donabedian A (1980). Explorations in quality assessment and monitoring: the definition of quality and approaches to its assessment.

[37] Martin DP, Diehr P, Price KF, Richardson WC (1989). Effect of a gatekeeper plan on health services use and charges: a randomized trial. Am J Public Health.

[38] Kerr EA, Hays RD, Mitchinson A, Lee M, Siu AL (1999). The influence of gatekeeping and utilization review on patient satisfaction. J Gen Intern Med.

[39] Yang H, Shi L, Lebrun LA, Zhou X, Liu J, Wang H (2013). Development of the Chinese primary care assessment tool: data quality and measurement properties. Int J Qual Health Care.

[40] Shi L, Starfield B, Xu J (2001). Validating the adult primary care assessment tool. J Fam Pract.

[41] Macinko J, Almeida C, de Sa PK (2007). A rapid assessment methodology for the evaluation of primary care organization and performance in Brazil. Health Policy Plan.

[42] Lee JH, Choi YJ, Sung NJ, Kim SY, Chung SH, Kim J (2009). Development of the Korean primary care assessment tool--measuring user experience: tests of data quality and measurement performance. Int J Qual Health Care.

[43] Mola E (2013). Patient empowerment, an additional characteristic of the European definitions of general practice/family medicine. Eur J Gen Pract.

[44] Wei X, Yin J, Wong SY, Griffiths SM, Zou G, Shi L (2017). Private ownership of primary care providers associated with patient perceived quality of care: a comparative cross-sectional survey in three big Chinese cities. Medicine (Baltimore).

[45] Chen W, Zhang Q, Renzaho AMN, Zhou F, Zhang H, Ling L (2017). Social health insurance coverage and financial protection among rural-to-urban internal migrants in China: evidence from a nationally representative cross-sectional study. BMJ Glob Health.

[46] Rask KJ, Deaton C, Culler SD, Kohler SA, Morris DC, Alexander WA (1999). The effect of primary care gatekeepers on the management of patients with chest pain. Am J Manag Care.

[47] Wang Y, Liu W, Wang X (2017). General practitioners in China during 2012—2015: development trend and distribution equity. Chinese General Practice.

[48] Isabel C, Paula V (2010). Geographic distribution of physicians in Portugal. Eur J Health Econ.

[49] Nuffield Trust. Number of general practitioners per 1,000 population. London; 2014. https://www.nuffieldtrust.org.uk/chart/number-of-general-practitioners-per-1-000-population. Accessed 25 June 2019.

[50] Centers for Disease Control and Prevention. State Variability in Supply of Office-based Primary Care Providers: United States, 2012. National Center for Health Statistics. 2014. https://www.cdc.gov/nchs/products/databriefs/db151.htm. Accessed 25 June 2019.

[51] Boerma WG, van der Zee J, Fleming DM (1997). Service profiles of general practitioners in Europe. European GP task profile study. Br J Gen Pract.

[52] Forrest CB, Starfield B (1998). Entry into primary care and continuity: the effects of access. Am J Public Health.

[53] Halm EA, Causino N, Blumenthal D (1997). Is gatekeeping better than traditional care? A survey of physicians' attitudes. Jama..

[54] Grumbach K, Selby JV, Damberg C, Bindman AB, Quesenberry C, Truman A (1999). Resolving the gatekeeper conundrum: what patients value in primary care and referrals to specialists. Jama..

[55] Shi L, Forrest CB, Von Schrader S, Ng J (2003). Vulnerability and the patient-practitioner relationship: the roles of gatekeeping and primary care performance. Am J Public Health.

[56] Hudon C, Fortin M, Haggerty JL, Lambert M, Poitras ME (2011). Measuring patients' perceptions of patient-centered care: a systematic review of tools for family medicine. Ann Fam Med.

[57] Greenfield G, Foley K, Majeed A (2016). Rethinking primary care's gatekeeper role. BMJ..

